# DNA Damage-Mediated Neurotoxicity in Parkinson’s Disease

**DOI:** 10.3390/ijms24076313

**Published:** 2023-03-28

**Authors:** Zhong-Xuan Wang, Yao-Lin Li, Jia-Li Pu, Bao-Rong Zhang

**Affiliations:** Department of Neurology, Second Affiliated Hospital, School of Medicine, Zhejiang University, Hangzhou 310009, China; 22018220@zju.edu.cn (Z.-X.W.); lylllfs@163.com (Y.-L.L.)

**Keywords:** Parkinson’s disease, DNA damage, DNA repair, neurodegeneration

## Abstract

Parkinson’s disease (PD) is the second most common neurodegenerative disease around the world; however, its pathogenesis remains unclear so far. Recent advances have shown that DNA damage and repair deficiency play an important role in the pathophysiology of PD. There is growing evidence suggesting that DNA damage is involved in the propagation of cellular damage in PD, leading to neuropathology under different conditions. Here, we reviewed the current work on DNA damage repair in PD. First, we outlined the evidence and causes of DNA damage in PD. Second, we described the potential pathways by which DNA damage mediates neurotoxicity in PD and discussed the precise mechanisms that drive these processes by DNA damage. In addition, we looked ahead to the potential interventions targeting DNA damage and repair. Finally, based on the current status of research, key problems that need to be addressed in future research were proposed.

## 1. Introduction

Parkinson’s disease (PD) is a very common neurodegenerative disease characterized by the selective death of dopaminergic (DA) neurons in the substantia nigra and the formation of Lewy bodies (LB) [1,2]. Clinically, PD mainly manifests as motor symptoms, such as bradykinesia, resting tremor, myotonia, and gait and postural abnormalities, as well as non-motor symptoms, such as hyposmia, sleep disturbance, autonomic dysfunction, and depression [3,4]. Globally, the prevalence of PD has increased significantly over the past three decades [1]. This disease not only severely affects the quality of life of patients, but also imposes a heavy emotional and financial burden on families and society [1,5]. At present, the treatment of PD is mainly symptomatic, which cannot completely solve the fundamental pathological problem of the selective death of DA neurons [6,7]. Therefore, it is necessary to further explore the pathogenesis of PD in order to find more optimal treatment options.

A well-established link between DNA damage and neurodegeneration has long existed [8,9]. Evidence for a causal contribution of DNA damage in the onset and progression of neurodegenerative diseases comes from rare patients and model organisms with mutations in DNA damage-responsive genes [10,11]. Interestingly, increasing evidence suggests that DNA damage and repair deficiency are also implicated in age-related neurodegenerative diseases, such as AD (Alzheimer’s disease), PD, ALS (Amyotrophic lateral sclerosis), and Huntington’s disease (HD) [12,13,14,15,16,17]. In PD, the pathogenicity of DNA damage is not only enhanced by its association with typical PD pathology, but also by the fact that DNA damage accumulation occurs before the onset of PD, implying its direct involvement in the pathophysiology [18,19,20]. More importantly, the loss of function of specific DNA repair proteins in mice can recapitulate part of the disease phenotype of PD, and some proteins known to play key roles in PD have been shown to directly mediate DNA repair [14,21,22,23,24,25]. All this evidence suggests that DNA damage may be the direct mechanism of neurodegeneration in PD. In this review, we summarized the causes of DNA damage in PD and the evidence for DNA damage-mediated neurotoxicity, and elucidated the relevant mechanisms. In addition, we analyzed the value of DNA damage repair as a potential therapeutic target for PD. Finally, we discussed the existing problems and future research directions in this field.

## 2. Evidence for DNA Damage-Mediated Neurodegeneration in PD

In fact, evidence of DNA damage in PD was reported several decades ago [14]. Compared with healthy controls, the level of 8-OHdG, a marker of oxidative DNA damage, was significantly upregulated in the DA neurons of PD patients [26]. Moreover, there were also much higher levels of 8-OHdG in the plasma, urine, and cerebrospinal fluid of PD patients than in controls [27]. In addition to oxidative damage, numerous studies have also found a significant upregulation of γ-H2AX, a hallmark of DNA double-strand breaks (DSBs) [18,28], in PD. Notably, this type of damage not only covers DA neurons, but also microglia [28]. To date, DNA damage-mediated neurotoxicity has been demonstrated in various toxin models of PD [15]. For example, both 8-OHdG and γ-H2AX can be induced by PD-associated neurotoxins, including 1-methyl-4-phenyl-1,2,3,6-tetrahydropyridine (MPTP), rotenone, paraquat, and 6-hydroxydopamine (6-OHDA) [28,29,30,31,32,33]. What is more, DNA damage-dependent activation of downstream signaling contributed to the degeneration and death of DA neurons in PD mouse models [34,35,36,37,38], suggesting that DNA damage may be an important link in the pathogenesis of sporadic PD. Moreover, studies of genetic defects and disease-associated proteins have provided us with a window to understand the role of DNA damage in the pathogenesis of PD [8]. So far, more than 20 causative genes were reported to contribute to familial PD, including *SNCA*, *LRRK2*, *PRKN*, *PINK1*, and *DJ-1* [39]. The first identified PD gene was *SNCA*, which encodes the protein α-synuclein, a key component of LB, whose overproduction increases the risk of PD [40]. Recently, Milanese et al. reported evidence of DNA damage accumulation, as well as the activation of the DNA damage response (DDR), in two PD mouse models based on the AAV-mediated overexpression of α-synuclein and α-synuclein pre-formed fibrils (PFF), respectively [8]. In this study, the accumulation of DSBs preceded the onset of the motor phenotype and DA degeneration, indicating that DSBs may have been the initiating lesion of neurotoxicity in these mice [8]. *LRRK2* is the most common causative gene for PD, and its role in genome stability has been recently emphasized [41,42]. It has been reported that LRRK2 protein can be involved in DDR, while LRRK2 deficiency exacerbates the age-dependent accumulation of DSBs in neurons [41,43]. Consistently, the DDR pathway was found to be activated in the LRRK2 G2019S model in vitro [44]. Mutations in *PRKN*, which encodes the protein parkin, are the most frequent cause of autosomal recessive early-onset PD [45]. Parkin deficiency could also lead to upregulation of oxidative DNA damage levels in the mouse brain [46].

It has to be mentioned that mutations in several genes encoding key proteins involved in DNA repair have been shown to contribute to the progression of PD [14]. For instance, mice lacking the DNA repair protein ATM exhibited PD-like motor abnormalities, accompanied by reduced expression of tyrosine hydroxylase (TH) and the dopamine transporter (DAT), as well as α-synuclein-positive inclusions [21,47]. ATM is an essential part of double-stranded break repair (DSBR), and without it, the balance between DNA damage and repair will be lost, leading to DNA damage accumulation [48]. Furthermore, mice lacking OGG1, a DNA repair enzyme for oxidative DNA damage, have been shown to exhibit age-related loss of DA neurons, the formation of ubiquitin-positive inclusions, and spontaneous motor behavior deficiencies, and to be more susceptible to MPTP-induced damage [24,49]. In addition, Sepe and colleagues reported the importance of ERCC1, a critical player in nucleotide excision repair (NER), in the preservation of the DA system [23,50]. They found that ERCC1 mutant mice exhibit typical PD-like pathological changes, including increased phospho-α-synuclein levels, reduced expression of TH-positive neurons, increased astrocyte activation, and mitochondrial dysfunction [23]. ERCC1 mutant mice are also more sensitive to the neurotoxin MPTP [23]. Recently, Miner et al. found that the loss of APE1 expression amplifies the formation of α-synuclein inclusions in vitro [51]. APE1 is a key enzyme in the base excision repair (BER) pathway, which mainly repairs oxidative DNA damage and the alkylation of bases [52]. Thus, the above evidence suggests that DNA damage can specifically affect the DA system and is closely associated with PD pathology.

## 3. Cause of DNA Damage in PD

**Figure 1:**
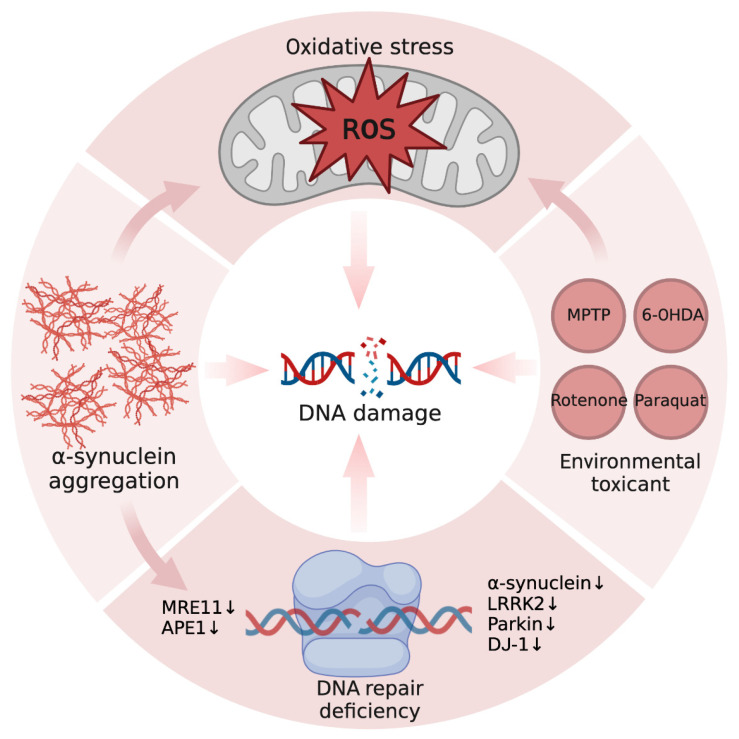
Sources of DNA damage in PD. Reactive oxygen species (ROS) can directly attack genomic DNA, causing many types of DNA damage. Pathogenic α-synuclein can damage DNA indirectly through ROS or directly cleave DNA, leading to DNA strand breakage. On the other hand, α-synuclein can also cause DNA repair defects by affecting the expression of DNA repair proteins, including MRE11 and APE1. Recent studies have highlighted the role of PD-causing proteins in DNA repair, including endogenous α-synuclein, LRRK2, parkin, and DJ-1, which partially explains the cause of abnormal DNA repair and damage accumulation in PD. Finally, environmental toxicants, an important component in the pathogenesis of sporadic PD, can also induce genomic DNA damage and lead to DNA damage-dependent dopaminergic neuron death. The pink arrows in the figure represent causal effects, and the black arrows indicate downregulation of protein expression. (Created with BioRender.com).

Although the phenotypes of DNA damage in PD are well defined, the reasons behind them are still unclear. Here, we briefly summarize the relevant mechanisms (Figure 1).

### 3.1. Oxidative Stress

Oxidative stress is a serious imbalance between the production of reactive oxygen species (ROS) and antioxidant defenses, leading to cellular damage and eventually death [53]. ROS are highly reactive free radical oxidants, including superoxide anion (O2-), hydrogen peroxide (H2O2), and hydroxyl radicals (OH·) [54]. It has been found that ROS can attack all macromolecules, including lipids, proteins, and nucleic acids, causing a defect in their physiological function [53]. ROS can lead to many different types of DNA damage, for example, direct modification of nucleotide bases, formation of apurinic/apyrimidinic (AP) sites, single-strand breaks (SSBs), and, less commonly, DSBs [55,56]. The most common oxidative DNA lesion is 8-OHdG, which is generated by the attack of OH· at the C-8 position of guanine [57]. Compared to other organs, the brain is considered particularly vulnerable to the devastating effects of ROS due to its high metabolic rate and relatively low capacity for cellular regeneration [58]. Consistently, oxidative stress has been considered as the main event leading to the degeneration of DA neurons in PD [59]. As previously mentioned, elevated levels of oxidative DNA damage have been reported in the brains of PD patients [26,27]. Further evidence linking oxidative stress to PD pathogenesis has been provided by animal models induced by neurotoxins that lead to ROS production, DNA damage, and progressive loss of DA neurons [60,61]. Similarly, functional studies of proteins associated with PD-causing genes have also been found to be related to the disruption of redox balance [62]. For instance, pathogenic α-synuclein aggregates localize to mitochondria and compromise respiratory chain components, resulting in mitochondrial dysfunction, massive production of harmful ROS, and DNA damage that can be prevented by the antioxidants N-acetylcysteine (NAC) and nitric oxide synthase (NOS) inhibitor [18,63].

### 3.2. Protein Aggregation

Loss of protein homeostasis is the central event in the pathogenesis of many neurodegenerative diseases [64,65]. Safeguarding protein homeostasis is essential for all cells, as proteome instability will lead to proteotoxic stress and protein aggregation, which in turn drives dysregulation and functional impairment of cellular pathways, resulting in cell degeneration and death [65,66]. Recently, several studies have reported that protein aggregation may be a cause of DNA damage [65,67]. Protein aggregation can cause DNA damage directly or indirectly through oxidative stress [65]. It has been reported that protein aggregates, including α-synuclein, can trigger oxidative stress, causing oxidative DNA damage in neurons [18,19,68]. In addition, α-synuclein is also thought to have a direct effect on DNA integrity [69,70]. It has been found that α-synuclein can directly bind to nuclear DNA and cleave DNA through its chemical nuclease activity, causing DNA strand breaks in the neuronal genome [69].

The ability of DNA repair may also be affected by α-synuclein [25,71]. New studies show that the overexpression or pathogenic aggregation of α-synuclein can interfere with the DNA repair process [71]. In the SH-SY5Y cell model, α-synuclein overexpression can lead to reduced expression of MRE11 and DSBs [71]. It has been demonstrated that MRE11 forms an MRN complex with RAD50 and NBS1 to recognize DSBs, and to initiate the downstream repair process [72,73]. Therefore, a decrease in MRE11 may lead to DSBR defects [73]. In addition, the expression of the APE1 protein was reduced in the α-synuclein PFF mouse model compared to controls [51], and APE1 deficiency will result in the accumulation of oxidized DNA damage [74]. However, it is unclear whether the reduced levels of MRE11 or APE1 proteins directly contribute to the decreased efficiency of DSBR or BER in the PD model.

### 3.3. DNA Repair Deficiency

Although the evidence of DNA damage in PD has long been reported, the mechanisms of DNA repair in PD are only beginning to be understood [75]. Unlike other age-related neurodegenerative diseases, many DNA repair proteins are upregulated in the brains of PD patients and animal models, which is partially explained by DNA damage-induced repair activation [15,75]. Conversely, a growing body of studies suggests that DNA repair is defective in PD [14,15]. For example, the ability of NER was significantly reduced in skin fibroblasts isolated from patients with PD [23]. Furthermore, some PD-causing proteins have been shown to regulate DNA repair [14]. As mentioned above, under physiological conditions, α-synuclein is not only enriched within the synapse, but a portion of α-synuclein is also distributed in the nucleus [76]. Although the role of α-synuclein in the nucleus has been controversial, recent work has identified a protective role for nuclear α-synuclein in DNA repair [25]. Specifically, endogenous α-synuclein promotes DSBR via nonhomologous end joining (NHEJ), implying that cytoplasmic aggregation of α-synuclein may gain toxicity by causing the loss of function of nuclear α-synuclein [25]. Similarly, a role for wild-type LRRK2 in DNA repair has recently been identified [44]. Endogenous LRRK2 responds to DNA damage and promotes homologous recombination (HR) repair, whereas its deficiency leads to DNA damage accumulation and loss of nuclear structure integrity [41,44,77]. In terms of autosomal recessive pathogenic proteins, parkin has been reported to also protect nuclear DNA and regulate NER [46,78]. In addition, DJ-1 is also reported to interact with XRCC4, a key component of NHEJ repair, in lung tissue, suggesting that DJ-1 may be implicated in the DNA repair process [79,80]. Thus, this evidence suggests that the accumulation of DNA damage and defective DNA repair in PD is mediated, at least in part, by the mutation-induced loss of function of these pathogenic proteins.

### 3.4. Environmental Toxicant

For a long time, environmental toxins have been associated with the etiology of sporadic PD [81]. In addition to the conventional phenotypes of mitochondrial dysfunction and oxidative stress, genomic toxic stress is also a common feature of these environmental toxin models [82,83]. For example, 1-methyl-4-phenylpyridinium (MPP+) can induce cells to produce DSBs and rapidly activate ATM and its downstream effectors, leading to apoptosis [36]. Similar to MPP+, it has been shown that exposure to 6-OHDA can cause persistent activation of DDR, ultimately resulting in cell death [29,33]. Whether in vivo or in vitro models, rotenone has been reported to cause both oxidative DNA damage and DSBs [83,84]. Moreover, exposure to paraquat can also elicit genotoxic stress in post-mitotic DA neurons [28]. These findings provide the proof of concept that DNA damage is a shared pathway in the degenerative death of DA neurons caused by environmental toxins.

## 4. Mechanisms of DNA Damage-Mediated Neurotoxicity in PD

**Figure 2:**
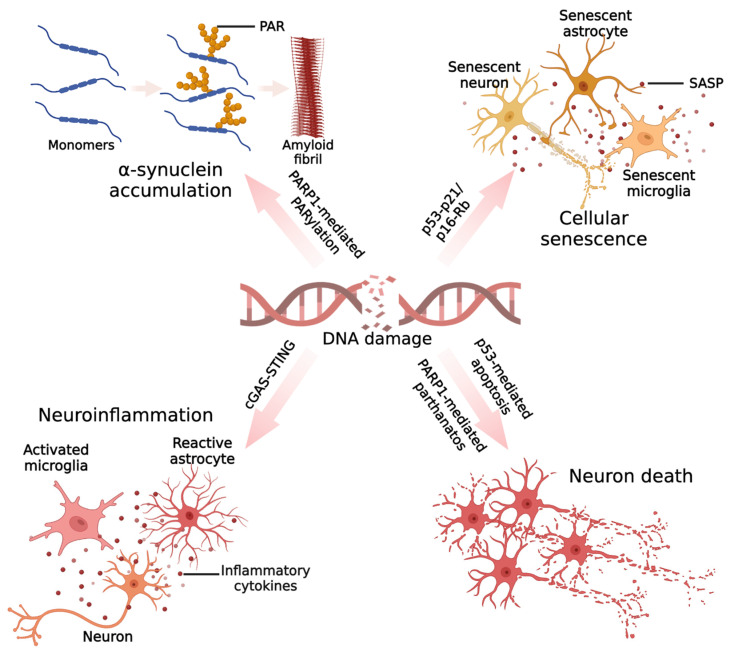
Mechanisms by which DNA damage could promote neurodegeneration in PD. In PD, the accumulated DNA damage leads to excessive activation of PARP1, and activated PARP1 can synthesize a large number of PAR chains. PAR is able to interact with the N-terminal portion of α-synuclein and promotes the fibrillation of α-synuclein monomers. Cellular senescence phenotypes have been reported in PD models, including senescent neurons, senescent microglia, and senescent astrocytes. In neurons, α-synuclein can induce DNA damage and upregulate the senescence markers p53 and p21. In microglia and astrocytes, pathogenic α-synuclein leads to the upregulation of P21 expression. In addition, the neurotoxin paraquat can cause astrocyte senescence phenotypes in vivo and in vitro, including DNA damage accumulation, p16 upregulation, and senescence-associated secretory phenotypes (SASPs). DNA damage has recently been found to induce inflammatory responses in several types of neuronal cells, including microglia, astrocytes, and neurons. The innate immune response induced by the cGAS–STING pathway plays a major role. Evidence for DNA damage-induced neuroinflammation in PD has been demonstrated in microglia. α-synuclein can attack the genomic DNA of microglia, which in turn activates the STING-dependent inflammatory response, leading to a massive release of inflammatory factors. The oxidative stress induced by inflammatory factors may further lead to DNA damage in other cells, contributing to the continued activation of the DNA damage response and causing a vicious cycle between DNA damage and inflammation. Lastly, DNA damage is thought to be an important cause of dopaminergic neuron death. Specifically, on one hand, DNA damage in PD models can activate a P53-dependent apoptotic program, and on the other hand, persistent DNA damage-induced hyperactivation of PARP1 can trigger parthanatos in neurons. (Created with BioRender.com).

In general, minor DNA damage can be repaired in the presence or absence of cell cycle arrest [85]. However, more intense and irreparable DNA damage can chronically activate DDR in neurons, leading to neuronal dysfunction and cell death [85,86,87]. Therefore, understanding how DNA damage contributes to neuronal loss at the cellular and molecular levels is critical. Below, we discuss recent mechanistic insights into the involvement of DNA damage in neurodegeneration in PD (Figure 2).

### 4.1. Protein Aggregation

Aggregation of specific proteins in disease-specific patterns of the brain is a notable feature of age-related neurodegenerative diseases, including PD, although the exact mechanisms remain unclear [88,89]. Interestingly, recent studies have found that DNA damage is also a crucial contributor to protein aggregation in neurodegeneration [90,91]. Lee et al. found that the accumulation of DNA damage caused by ATM loss can lead to extensive protein aggregation. DNA damage-induced hyperactivation of PARP1 is thought to drive protein aggregation in ATM-deficient models [90]. To be specific, PARP1-mediated poly-ADP-ribosylation (PARylation) is involved in protein aggregation in ATM-deficient cells by attracting intrinsically disordered proteins [90]. Moreover, the study by Wouter et al. also found that genomic toxicity induced by targeting ATM, ATR, or DNA topoisomerase can trigger the extensive aggregation of proteins with a propensity for liquid–liquid phase separation (LLPS), which can also be mitigated by the inhibition of PARylation [92]. In ALS, numerous studies have shown that PAR-mediated LLPS facilitates the fibrillation of aggregation-prone proteins and their maturation into insoluble precipitates over time [93,94,95]. For instance, PAR has been shown to promote LLPS of FUS, TDP43, and hnRNPA1 in both ex vivo and cellular settings, while increased concentrations of ALS-related proteins in droplets is able to promote their own pathological fibrosis [96,97,98,99]. Recent evidence has also demonstrated that α-synuclein is an intrinsically disordered protein and has the capacity to condense into liquid-like droplets through LLPS in ex vivo and in vitro cell lines [100,101,102]. Interestingly, PAR also significantly promotes amyloid aggregation of α-synuclein [19]. Mechanistically, PAR interacts directly with α-synuclein and accelerates the pathogenic aggregation and toxicity of α-synuclein in vivo and in vitro [19]. Thus, these results suggest that increased PAR levels due to DNA damage can act as a nucleation center to promote the irreversible aggregation of intrinsically disordered proteins, thereby amplifying neurotoxicity and driving neurodegeneration [103,104,105].

### 4.2. Cellular Senescence

Cellular senescence is a state of irreversible cell cycle arrest [106]. Currently, cellular senescence is considered as a stress response that can be induced by various exogenous or endogenous stresses, such as genotoxic telomere shortening, replicative stress, and oncogene activation [107]. While cellular senescence is caused by multiple factors, persistent DDR is recognized as the primary mechanism for establishing and maintaining the senescent phenotype [108]. In vitro, a variety of DNA-damaging agents have been used to induce cellular senescence, including radiation (ionizing radiation and ultraviolet light) and chemotherapeutic agents (etoposide, bleomycin, and doxorubicin) [109,110]. In terms of mechanisms, DDR activates the p16-Rb and p53-p21 pathways to impede the cell cycle and execute the senescence program [87,108]. After DNA damage, activated p53 promotes transcription of p21, which blocks CDK2 activity, leading to Rb hypophosphorylation and cell cycle exit [110,111]. On the other hand, p16 inhibits CDK4/6, thereby preventing the phosphorylation of Rb and the progression of the cell cycle [110,111]. In addition, most senescent cells can develop a senescence-associated secretory phenotype (SASP), consisting of growth factors, extracellular matrix components, interleukins, chemokines, metalloproteinases, and other signaling molecules [112]. Persistent activation of DDR is critical for the initiation and maintenance of SASP, whereas the inflammation-associated transcription factor NF-κB is a key player in the regulation of SASP [113,114,115]. The primary function of SASP is to promote the repair of damaged tissues and to recruit immune cells to remove senescent cells, thereby restoring normal tissue function [111]. Unfortunately, long-term exposure to SASP can lead to chronic inflammation and disease [111,116]. It has been shown that numerous cell types in the brain, including neurons, microglia, astrocytes, and oligodendrocytes, are able to activate the cellular senescence program [117]. Remarkably, such senescent cells are closely associated with the disease progression of age-related neurodegenerative diseases, such as AD and PD [106,117,118]. It has been reported that PD patients have higher expression of senescence and SASP markers, such as p16, MMP-3, IL-6, IL-1α, and IL-8, in body fluids and brain tissue compared to healthy controls [119,120]. Recent evidence also shows that pathogenic α-synuclein is sufficient to induce DNA damage and cellular senescence in several brain cell types [71,121,122]. Furthermore, cellular senescence can be triggered in vitro and in vivo by exposure to paraquat and MPTP, while selective elimination of senescent cells can reduce paraquat-induced neurotoxicity, indicating that cellular senescence contributes to DA neurodegeneration [121,123]. However, it is not yet clear whether DNA damage is the primary inducer of the senescence phenotype in these cell types.

### 4.3. Neuroinflammation

Neuroinflammation is one of the pathological features of PD [124]. The chronic inflammatory state observed in PD models is thought to be responsible for the subsequent neuronal loss [125]. Therefore, elucidating the potential sources and molecular mechanisms of inflammation has become a key scientific question in the field of PD research [126]. Emerging evidence suggests that DNA damage is able to induce inflammation in vivo and in vitro [127]. In the central nervous system (CNS), it has been reported that DNA damage can induce inflammatory responses in multiple neural cell types, which together impact neural homeostasis [128]. Below, we discuss the latest mechanistic insights between DNA damage and neuroinflammation in microglia, astrocytes, and neurons and how their interaction influences neurodegeneration in the state of DNA damage.

#### 4.3.1. Microglia

For a long time, abundant evidence supported the role of microglia in the pathogenesis of PD [126,129]. Activated microglia can exacerbate neurodegeneration by creating a cytotoxic environment for neurons, yet the specific factors contributing to microglia activation are not well understood [129,130]. It has been shown that DNA damage can activate microglia and lead to the secretion of pro-inflammatory cytokines, causing severe damage to neuronal cells [131,132,133]. This phenotype is primarily induced by activation of the damaged DNA-driven cGAS–STING signaling pathway, which is thought to initiate germ-free inflammation through both type-I interferon and NF-κB signaling [131,133]. For example, DNA damage in microglia directly induced by etoposide can trigger elevated cytoplasmic DNA levels, which in turn stimulates an antiviral innate immune response via the cytoplasmic DNA sensor STING, ultimately rendering microglia to exhibit a neurotoxic proinflammatory phenotype [131]. In addition, DNA damage accumulation due to the defective DNA repair associated with the ATM deficiency in microglia can also trigger pro-inflammatory and neurotoxic signals by activating the cGAS–STING pathway [133]. Recently, DNA damage-induced microglial activation was also observed in PD [134]. Hinkle et al. demonstrated that misfolded α-synuclein can cause genomic DNA damage in microglia and, thus, trigger STING-dependent microglial inflammation [134]. More importantly, STING knockout reduced neuroinflammation and DA neuron degeneration in an α-synuclein PFF model [134], suggesting the pathogenic significance of DNA damage in mediating microglia–neuron interactions.

#### 4.3.2. Astrocytes

The neurotoxic activity of astrocytes has been reported in PD studies [135]. Interestingly, DNA damage has been also found to contribute to neurotoxic inflammation in astrocytes [136,137]. For instance, DNA damage induced directly by etoposide or indirectly through the inhibition of ATM can lead to the accumulation of cytosolic DNA and STING-associated inflammatory responses in astrocytes, causing the secretion of neurotoxic mediators and neuronal atrophy and death [137]. In addition, DNA damage as a driver of neurotoxic inflammation in astrocytes was recently demonstrated in Aicardi–Goutières syndrome (AGS), an inherited encephalopathy [136]. In this study, p53-dependent DDR was thought to contribute to AGS astrocyte-mediated neurotoxicity [136]. In PD, exposure to paraquat has been found to cause DNA damage in astrocytes, and conditioned medium from paraquat-treated astrocytes significantly reduced the viability of DA neurons [123]. Although there is no direct evidence for the role of DNA damage in astrocytic neurotoxicity in PD, considering the potential damaging effects of inflammatory factors and pathogenic α-synuclein on astrocytic genomic DNA [128,135,138], it is conceivable that DNA damage may be an important part of astrocytic activation and toxic amplification in PD. 

#### 4.3.3. Neurons

Surprisingly, in addition to glial cells, recent evidence suggests that DNA damage in neurons is also an important source of brain inflammation [11,139]. Welch et al. found that DNA damage in neurons can induce senescence and antiviral-like signals that promote neuroinflammation by secreting chemotactic and pro-inflammatory cytokines to attract microglia [139]. Although the innate immune response triggered by the cGAS–STING pathway occurs primarily in glial cells, work investigating neurotropic viral infections has shown that neuron-derived inflammatory signaling is also a key feature of the antiviral response [11]. Notably, neuronal cGAS–STING-mediated inflammatory responses have been demonstrated in HD and ALS models, indicating that inflammation in neurons burdened with DNA damage plays an important role in neurodegeneration [140,141]. In addition, inflammatory factors secreted by glial cells can also cause DNA damage in the neuronal genome [128,136], thus contributing to the continued activation of DDR, forming a vicious cycle, and ultimately exacerbating the neurodegenerative process. As mentioned earlier, the pathogenicity of DNA damage has been confirmed in DA neurons in PD [11]; however, it remains unknown whether the process of DNA damage-driven neurodegeneration in DA neurons involves inflammation.

### 4.4. Cell Death

The degeneration and death of DA neurons are the pathological basis of PD [142]. Numerous studies have shown that multiple types of programmed cell death (PCD) participate in the degenerative loss of DA neurons, including apoptosis, autophagy, necroptosis, parthanatos, and ferroptosis [143]. Here, we focus on apoptosis and parthanatos, both of which are closely related to DNA damage [144].

#### 4.4.1. Apoptosis

Apoptosis is the most investigated and best-known form of PCD [144,145]. The execution of apoptosis can be instigated by extrinsic or intrinsic signals. In the extrinsic pathway, apoptosis is activated by the binding of death receptors to extracellular ligands [144,145]. The intrinsic apoptosis pathway is induced by various microenvironmental perturbations, including (but not limited to) ROS, DNA damage, endoplasmic reticulum stress, and growth factor depletion [144,145]. Both pathways can alter the permeability of the inner mitochondrial membrane, ultimately causing the release of pro-apoptotic factors from the mitochondria into the cytoplasm, such as apoptosis-inducing factor (AIF), cytochrome c, Smac/Diablo, Omi/HtrA2, and endonuclease G [144,145]. These factors then facilitate the execution of apoptosis in a caspase-dependent or caspase-independent manner [144,145]. In the context of PD, there is abundant evidence from both patients and experimental models supporting the role of DNA damage-triggered intrinsic apoptotic pathways in DA neurodegeneration [146,147]. For example, upregulation of DNA damage and p53, a powerful pro-apoptotic factor, has been reported in a MPTP-induced mouse model, and p53 inhibition can prevent neurodegeneration and ameliorate motor deficits [148,149]. Similarly, DNA damage and apoptotic phenotypes have been observed in other PD toxin models, as well as transgenic mouse models of PD, although p53 may not always be the mediator of cell death in these paradigms [29,147,150,151,152].

#### 4.4.2. Parthanatos

Parthanatos, a portmanteau of “par” (for PAR polymer) and “thanatos” (for death in Greek mythology), was first named in 2008 by Dawson’s team at Johns Hopkins University as a new model of PCD [38,144]. Parthanatos is broadly characterized by five biochemical stages: DNA damage, PARP1 hyperactivation, PAR association with AIF, AIF release and MIF/AIF complex translocation, and MIF-mediated massive DNA breaks [38,153]. More specifically, various toxic stimuli cause persistent DNA damage and hyperactivation of PARP1, which synthesizes large amounts of long-chain, branching PAR polymers by consuming NAD and ATP [154,155,156]. Subsequently, PAR moves from the nucleus to the cytoplasm and associates with AIF in the mitochondria, leading to the release of AIF into the cytoplasm [157,158,159]. AIF then interacts with MIF and recruits MIF to the nucleus to cleave DNA, causing massive DNA breaks and eventually cell death [157,158,160,161]. Parthanatos is widely present in different diseases, including cardiovascular diseases, renal diseases, diabetes mellitus, cerebral ischemia, and neurodegenerative diseases [162,163,164]. Parthanatos has been extensively studied and confirmed in PD for more than two decades [19,165,166]. Back in 1999, Dawson’s team published the first report related to PARP1-mediated neurodegeneration in a mouse model of PD [167]. In this study, they found that PARP1 activation is involved in MPTP-induced neurotoxicity and demonstrated that nitric oxide (NO)-induced DNA damage is necessary for PARP1 activation [167]. In two recent studies, their group further identified the key role of parthanatos in α-synuclein toxicity [19,166]. They found that α-synuclein PFF can cause DNA damage by triggering NO production, thereby inducing excessive activation of PARP1 and eventually leading to cell death via parthanatos [19].

## 5. Developing Interventions through DNA Damage and Repair

**Figure 3:**
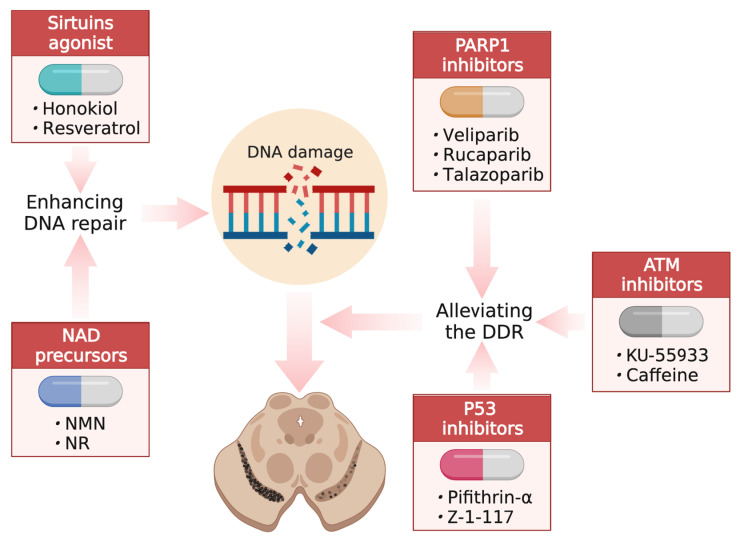
Therapeutic options for targeting DNA repair or DDR in PD. An overview of current compounds that target DNA repair or DDR in PD. Abbreviations: DDR, DNA damage response; NMN, nicotinamide mononucleotide; NR, nicotinamide riboside. (Created with BioRender.com).

In light of the potential role of DNA damage in PD and other age-related neurodegenerative diseases, the interventions that target DNA damage and repair are a major emerging focus in this field [8,168,169]. There are two main approaches currently under development, including enhancing DNA repair and alleviating the DDR [168,170,171,172,173,174] (Figure 3 and Table 1).

### 5.1. Enhancing DNA Repair

Whether directly targeting DNA repair can delay the onset and progression of PD is an interesting topic. Indeed, enhancing DNA repair has been shown to improve neuronal survival after injury [175]. However, enhancing DNA repair is actually a huge challenge due to the intricacies of repair mechanisms [176]. There is already evidence that overexpression of DNA repair factors directly may have detrimental consequences [169,177]. Nevertheless, various drugs have been developed to directly or indirectly increase DNA repair [169]. For example, numerous studies have confirmed that increasing NAD levels is a promising strategy for stimulating DNA repair and preventing neurodegeneration [178,179,180,181]. Supplementation with NAD and its precursors, like nicotinamide mononucleotide (NMN), nicotinamide riboside (NR), and nicotinamide (NAM), can promote NHEJ repair through increasing the levels of chromatin-bound Ku70 and DNA-PKcs [178,182]. It has been reported that treatment of AD mouse models with NR can alleviate several major features of AD, including Tau pathology, DNA damage, neuroinflammation, synaptic dysfunction, and cognitive impairment [171,172]. In the context of PD, evidence from cellular and animal, as well as clinical trial, studies suggests that increasing NAD levels has shown beneficial impacts and that the protective effect of NAD is at least partly mediated through DNA repair [183,184,185,186,187,188,189,190]. Moreover, most mammalian sirtuins can stimulate DNA repair and maintain genome stability, including SIRT1, SIRT2, SIRT3, SIRT6, and SIRT7 [169,191]. SIRT1 regulates BER through APE1 and XRCC1 [192,193], NER through XPA [194], mismatch repair (MMR) through MSH2 and MSH3 [195], and DSBR through Ku70 and WRN [196,197]. SIRT2 stimulates DSBR through ATRIP [198]. SIRT3 promotes DSBR through Rad52 and Ku70 [199,200]. SIRT6 regulates BER by interacting directly with MYH, APE1, and Rad9-Rad1-Hus1 (9-1-1) [201], and DSBR by stimulating PARP1 activity [202]. SIRT7 promotes DSBR by H3K18Ac deacetylation and H3K122 desuccinylation [203,204]. In recent years, many preclinical studies have examined the effects of sirtuins in PD cells and animal models [205]. In this regard, it has been shown that the use of the SIRT1 agonist resveratrol and the SIRT3 agonist honokiol can improve the disease phenotype in PD mouse models [205,206,207,208,209], suggesting that promoting DNA repair is a potential target for PD therapy.

### 5.2. Alleviating the DDR

Unrepaired DNA damage can cause persistent activation of DDR and subsequent downstream signaling, ultimately leading to cellular senescence and death [86,170,210]. Indeed, in most cases, neurodegenerative diseases seem to arise through excessive activation of DDR, so drugs that suppress the DDR pathway can be used to treat these diseases [170]. As mentioned above, persistent DNA damage can lead to excessive PARP1 activation [19]. Activated PARP1 not only leads to bioenergetic deficiency through NAD depletion, but also induces parthanatos [19,156]. More importantly, PARP1 activation promotes amyloid aggregation and toxicity, while PARP1 inhibition can significantly alleviate the disease symptoms in PD, as well as other neurodegenerative disease models [19,38], indicating that the downregulation of the response to DNA damage may be beneficial. Several PARP1 inhibitors have been approved by the FDA for the treatment of various cancers [38]. Among them, PARP inhibitors (veliparib, rucaparib, and talazoparib) have been shown to prevent α-synuclein PFF-mediated neurotoxicity in both cellular and mouse models [19]. Notably, current PARP1 inhibitors have been shown to induce PARP1 capture on chromatin, thereby driving subsequent DNA damage, innate immune responses, and cytotoxicity, making them unsuitable as disease-modifying drugs for the treatment of neurodegeneration [38].

ATM is a major regulator of DDR, and it also serves a critical role in triggering cell death in response to genotoxic stress [48]. Persistent activation of ATM signaling has been demonstrated in mouse models of both PD and other neurodegenerative diseases, while targeted inhibition of ATM activity has neuroprotective effects [18,36,173]. For instance, Lu et al. reported evidence of the persistent activation of DDR in the brain tissue of HD patients and mice, and demonstrated that targeting ATM by gene knockout and pharmacological inhibition can ameliorate mutant Huntingtin (mHTT) toxicity in vivo and in vitro [173]. Caffeine, a non-specific inhibitor of ATM, was shown to protect neurons from etoposide-induced DNA damage and cell death in vitro [211]. In PD, inhibition of ATM by caffeine prevented 6-OHDA-induced apoptosis in DA neurons [212]. In another study, KU-55933, a selective ATM inhibitor, displayed a neuroprotective effect against MPP+-induced apoptosis in neuronal cells [36]. The above evidence suggests that targeting ATM is a useful therapeutic strategy in PD. However, it should be noted that ATM has over 700 potential targets, and, in addition to DDR, it regulates several downstream cellular processes [48,174]. It is imaginable that ATM inhibition may elicit certain side effects, which makes it a difficult therapeutic target [173,174].

In addition, various DNA damage agents, as well as the deficiency of essential DNA repair proteins, are able to induce DNA damage and activate p53-dependent apoptotic signaling in neurons [86,213,214]. While p53-independent apoptotic mechanisms have been identified in neurons following DNA damage, in most situations, p53 activity is enhanced upon DNA damage, and inhibition of p53 can mitigate the neurotoxic effects of DNA-damaging insults [86,213,214]. Indeed, pharmacological inhibition of p53, via pifithrin-α (PFT-α) and Z-1-117 or genetic reduction of p53, has been shown to reverse neurodegeneration in multiple PD models, raising the potential of p53 as a therapeutic target for PD [148,149,215]. However, it should be noted that the role of p53 in the CNS is complicated and remains to be fully defined [216,217].

## 6. Conclusions and Perspectives

Over the past two decades, increasing evidence has suggested a potential contribution of genome stability to the onset and progression of neurodegenerative diseases [8,9]. Now there is clear evidence that DNA damage makes a significant contribution to the progression of PD [14,15]. DNA damage may contribute to PD through cell-autonomous mechanisms (e.g., protein aggregation, senescence, and cell death) and non-cell-autonomous mechanisms (e.g., neuroinflammation) [218,219]. Nevertheless, many key questions remain unanswered. As described previously, the CNS is a highly integrated network of neuron and glial cells [220,221]. However, to date, most studies have focused on the effects of DNA damage and repair defects on neuronal cell function and viability [220,221]. In fact, microglia also display DNA damage in the brain tissue of PD patients, as well as in animal models [28,134]. Yet, the molecular mechanisms of DNA repair in microglia, the consequences of persistent DNA damage, and the mechanisms by which DNA damage affects the neighboring neurons have not been fully elucidated. In addition, the latest research suggests that DNA damage in neurons can also induce inflammatory phenotypes, including SASP and type I interferon responses, which have been shown to exacerbate disease progression in multiple models of neurodegenerative disease [139,140]. Compared to other neurons, the inflammatory signaling capacity in DA neurons has received little attention [11]. Indeed, evidence of DNA damage accumulation in DA neurons has been found in the early stages of PD animal models [18,20], but the specific molecular events of DNA damage-mediated neurodegeneration need to be further explored. Last, but not least, since DNA damage can directly cause neurodegeneration, and nearly all age-related neurodegenerative diseases are associated with DNA damage [8], why does the disease manifest differently in different individuals?

In summary, based on the existing evidence, the significance of DNA damage and repair abnormalities is emerging in the field of PD, and clarifying the relationship between DNA damage and PD will open up new avenues for understanding the pathogenesis of PD and provide fertile ground for future drug development.

## Figures and Tables

**Table 1:** The application of drugs targeting DNA repair or DDR in the treatment of PD.

Target	Drugs	Models	Effects	References
NAD	NMN	Rotenone-treated PC12 cells	DNA damage ↓; energy metabolism ↑; apoptosis ↓; necrosis ↓.	[183,187,188]
	NAM	MPP+-treated SK-N-MC cells; α-synuclein transgenic drosophila	Mitochondrial function ↑; oxidative stress ↓; DNA damage ↓; motor function ↑.	[189]
		Parkin or Pink1 mutant drosophila	Mitochondrial function ↑; loss of dopaminergic neurons ↓.	[185,190]
	NR	GBA-PD iPSC neurons and fly models of PD	Mitochondrial function ↑; autophagy ↑; motor function ↑.	[184]
		Double-blinded phase I clinical in PD patients	Mitochondrial function ↑; lysosomal and proteasomal function ↑; inflammation ↓;	[186]
SIRT1	Resveratrol	MPTP/6-ODHA/Rotenone-induced PD rodent models; A53T α-synuclein mice	α-synuclein pathology ↓; TH ↑; inflammation ↓; mitochondrial function ↑; oxidative stress ↓; motor function ↑	[207,208]
SIRT3	Honokiol	6-OHDA-lesioned mice	Oxidative stress ↓; TH ↑; motor function ↑	[209]
PARP1	Veliparib Rucaparib Talazopari	α-synuclein PFF-treated primary neurons and mice	α-synuclein pathology ↓; TH ↑; parthanatos ↓; motor function ↑	[19]
ATM	KU-55933	MPP+-treated cerebellar granule cells	DNA damage ↓; apoptosis ↓	[36]
	Caffeine	6-OHDA-treated PC12 cells	DNA damage ↓; apoptosis ↓	[152]
p53	Pifithrin-α	Paraquat-treated SY5Y cells	Mitochondrial function ↑; DNA damage ↓; apoptosis ↓	[151]
	Z-1-117	MPTP-treated mice	TH ↑; apoptosis ↓; motor function ↑	[148]

Abbreviations: NMN, nicotinamide mononucleotide; NAM, nicotinamide; NR, nicotinamide riboside; iPSC, induced pluripotent stem cell; MPP+, 1-methyl-4-phenylpyridinium; TH, tyrosine hydroxylase; MPTP, 1-methyl-4-phenyl-1,2,3,6-tetrahydropyridine; 6-OHDA, 6-hydroxydopamine. The up arrows in the table represent up-regulation, and the down arrows indicate down-regulation.

## Data Availability

Not applicable.
